# Delivering actionable infodemic insights and recommendations for the COVID-19 pandemic response

**DOI:** 10.1093/eurpub/ckac129.645

**Published:** 2022-10-25

**Authors:** TD Purnat, A Ishizumi, B Yau, B White, C Bertrand-Ferrandis, S Briand, T Nguyen

**Affiliations:** Epidemic and Pandemic Preparedness and Prevention, WHO, Geneva, Switzerland

## Abstract

**Issue:**

The COVID-19 pandemic and current recovery efforts have been complicated by a parallel infodemic. The infodemic has manifested itself in the rapid spread of questions, concerns and misinformation that can affect population attitudes and behavior harmful to health -promoting stigma and discrediting science, non-recommended treatments and cures, politicizing health programs and eroding trust in health workers and health systems.

**Description:**

WHO's COVID-19 Pillar 2 (risk communication, community engagement and infodemic management) developed an integrated public health infodemic insights methodology for weekly analysis of social media, traditional media and other data sources to identify, categorize, and understand the key concerns and narratives expressed, and inform risk communication and response activities.

**Results:**

The infodemic characterization, integrated analysis and insights generation consisted of a 3-step mixed-methods approach. First, data was collected from publicly available social and news media and categorized into categories of conversations by a COVID-19 public health taxonomy. Second, the dataset was analyzed and compared week-on-week to identify changes in narratives and conversation sentiment. Third, the digital infodemic intelligence was reviewed by a group of subject matter experts and triangulated with other data sources to derive infodemic insights and provide recommendations for action for the week. The methodology has been applied to inform COVID-19 response, COVID-19 vaccine demand promotion, and preparing for mass gatherings or mass immunization campaigns.

**Lessons:**

The methodology for infodemic intelligence generation and integration has introduced evidence-based analytical practices for generation of infodemic insights and recommendations for action into the work of WHO. It must be further adapted for use by different health programmes and preparedness functions, and is described WHO Field Infodemiology Manual.

**Key messages:**

• Health authorities can use infodemic insights to respond to people’s concerns, questions and information deficits in a timely and effective manner.

• An evidence-based methodology has been developed and validated to generate infodemic insights and recommendations for action during an acute health event or emergency.

